# Will a new clade of SARS-CoV-2 imported into the community spark a fourth wave of the COVID-19 outbreak in Hong Kong?

**DOI:** 10.1080/22221751.2020.1851146

**Published:** 2020-11-25

**Authors:** Gilman Kit-Hang Siu, Lam-Kwong Lee, Kenneth Siu-Sing Leung, Jake Siu-Lun Leung, Timothy Ting-Leung Ng, Chloe Toi-Mei Chan, Kingsley King-Gee Tam, Hiu-Yin Lao, Alan Ka-Lun Wu, Miranda Chong-Yee Yau, Yvette Wai-Man Lai, Kitty Sau-Chun Fung, Sandy Ka-Yee Chau, Barry Kin-Chung Wong, Wing-Kin To, Kristine Luk, Alex Yat-Man Ho, Tak-Lun Que, Kam-Tong Yip, Wing Cheong Yam, David Ho-Keung Shum, Shea Ping Yip

**Affiliations:** a Department of Health Technology and Informatics, The Hong Kong Polytechnic University, Hung Hom, Hong Kong Special Administrative Region, People’s Republic of China; b Department of Microbiology, Li Ka Shing Faculty of Medicine, The University of Hong Kong, Pokfulam, Hong Kong Special Administrative Region, People’s Republic of China; c Department of Clinical Pathology, Pamela Youde Nethersole Eastern Hospital, Chai Wan, Hong Kong Special Administrative Region, People’s Republic of China; d Department of Clinical Pathology, United Christian Hospital, Kwun Tong, Hong Kong Special Administrative Region, People’s Republic of China; e Department of Pathology, Princess Margaret Hospital, Kwai Chung, Hong Kong Special Administrative Region, People’s Republic of China; f Department of Clinical Pathology, Tuen Mun Hospital, Tuen Mun, Hong Kong Special Administrative Region, People’s Republic of China; g Faculty of Health and Social Science, The Hong Kong Polytechnic University, Hung Hom, Hong Kong Special Administrative Region, People’s Republic of China

**Keywords:** COVID-19, SARS-CoV-2, whole genome sequencing, Phylogeny, outbreak

Hong Kong has already been hit by three waves of the coronavirus disease 2019 (COVID-19) as of October 2020. More than 120 people were infected in the first round, which was triggered mainly by tourists arriving from Mainland China. Our team analysed the SARS-CoV-2 genomes of 50 cases up to 28 February 2020, and identified that the majority of the locally acquired cases belonged to GISAID clade V, which is characterized by the mutation *Orf3a* G251V [1]. The second wave was attributed to imported cases of students and working people returning from Europe and North America. Over 640 people had contracted the virus between March and April 2020. Multiple GISAID clades of SARS-CoV-2 were involved, including clade S, clade L, and most importantly clade G which harboured the hallmark mutation D614G in the spike (*S*) protein gene [2].

The third wave started in early July when infections surged abruptly. The virus swept through the city, affecting care homes for the elderly and the disabled, public hospitals, detention facilities, wet markets, and a container terminal. The total number of cases had expanded five-fold from just over 1000 in early July to over 5000 as of 31 August 2020 [3]. To *et al.* conducted whole-genome sequencing for 50 cases, and identified that the majority of genomes from locally acquired cases belonged to two novel lineages within the GISAID clade GR. Both lineages were phylogenetically related to the cases from marine crew and aircrew who were exempted from quarantine [2].

Zero local case was finally recorded on 15 September 2020 [3]. The city then relaxed social-distancing measures, including doubling the maximum number of people who could gather in the public to four, and reopening venues such as bars, cinemas and karaoke lounges. After a few days of relative quiescence, a series of untraceable locally acquired cases re-appeared since 30 September 2020 cases [3]. From 30 September to 11 October (data cut-off), there were 51 locally acquired cases, in which 13 had unknown source(s) and 38 were epidemiologically linked to these 13 [3,4]. While the relapse of local cases was believed to be due to untraceable infections leftover in the community from the third wave after the recent easing of restrictions, an influx of 170 infected travellers had been reported between 1 September and 11 October, 2020 (Supplementary Figure 1 and Supplementary Table 1) [3].

This study aimed to determine viral genomic characteristics of locally acquired infections reported in October 2020 and to investigate their phylogenetic relationship with the cases in the previous three waves of local outbreak and the recent imported cases reported between September and October 2020. Whole-genome sequencing (WGS) of SARS-CoV-2 was conducted on respiratory specimens using Nanopore MinION (Oxford Nanopore Technologies, Oxford, UK) coupled with Artic Network nCoV-2019 novel coronavirus bioinformatics protocol [5]. Phylogenetic analysis was performed using PhyML v3.0 with maximum likelihood algorithm [6]. Detailed methodologies are provided in Supplemental Materials.

This study was approved by the Institutional Review Boards of The Hong Kong Polytechnic University (RSA20021) and the public hospitals involved (HKECREC-20200014;KCC/KEC-20200070;KWC-20200040;NTWC-20200038).

A total of 64 SARS-CoV-2-positive specimens collected from four public hospitals were selected for WGS in this study as they had higher SARS-CoV-2 viral load (PCR Ct values <20). These included 15 cases collected in the second wave of local outbreak (March–April), 25 in the third wave (July–August), eight locally acquired infections reported in October 2020, and 16 imported cases reported in September and October 2020. The sequences were submitted to the NCBI GenBank with assession numbers MW181702 – MW181765.

For phylogenetic analysis, we included an additional of 18 genomes which were reported by us in the first wave of local outbreak (January–February) (Supplementary Table 2) [1]. Epidemiological information of these cases was retrieved from the Centre for Health Protection (CHP) of the Department of Health [3]. We adopted the CHP case numbering system, which was based on the date of case confirmation.

Here we showed that the eight locally acquired cases in October shared highly similar genome and belonged to GISAID clade GH, which is characterized by 241c > t, 3037c > t, 14408c > t (*Orf1b* P314L), 23403a > g (*S* D614G), and 25,563g > t (*Orf3a* Q57H) [7]. In addition, these cases shared 13 additional common mutations that are rarely found in other sequences publicly available at GISAID, including 922g > a, 1947t > c (*Orf1a* V561A), 3431g > t (*Orf1a* V1056L), 5653t > c, 5950g > a, 6255c > t (*Orf1a* A1997V), 7504c > t, 18,877c > t, 22,444c > t, 24,175t > c, 26,060c > t (*Orf3a* T223I), 26,735c > t, and 28,854c > t (*N* S194L). Notably, the new genomes differ from those in the third wave by at least 22 nucleotides (Supplementary Figure 2). Based on our previous study [1], the average evolutionary rate of SARS-CoV-2 was 3.04 × 10^−3^ substitutions per site per year, meaning that the virus may accumulate about 7.6 nucleotide changes per month. The significant variations in the genomes might indicate that the new SARS-CoV-2 variants are unlikely to evolve from the strain causing the third-wave outbreak.

**Figure 1.**
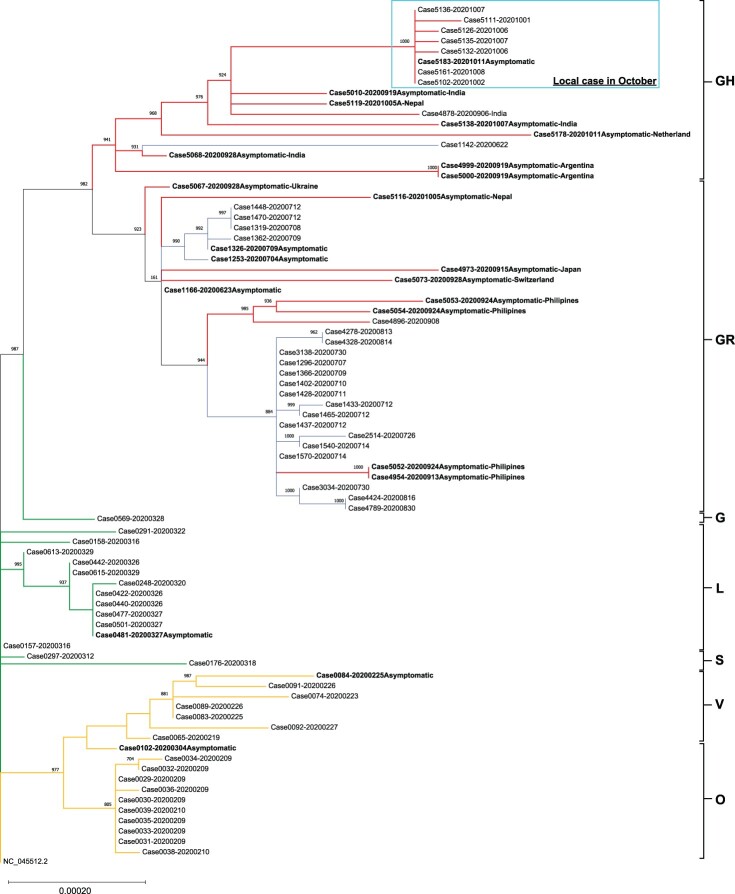
Phylogenetic tree constructed by PhyML using maximum likelihood with bootstrap value set at 1000×. The tree was rooted on the earliest published genome (accession no.: NC_045512.2). Each genomic sequence consisted of 29,782 bp. A total of 360 variable sites were identified by multiple alignment and were used for phylogenetic tree construction. Specimens were labelled by the CHP case number followed by the diagnostic date (YYYYMMDD). Asymptomatic patients were bolded and imported cases were labelled by the departing countries. The letters on the right of the figure are the GISAID phylogenetic clades. Branch lengths were measured in number of substitutions per site. The bar with 0.00020 means that there are approximately 6 substitutions (0.0002 × 29,903 bp) difference when two strains were located at the opposite end of a bar of this length. Branches were highlighted as follow: Orange for cases from January to February (genomes generated in our previous work [1]); Green for cases from March to April; Blue for cases from July to August; and Red for recent cases in September to October (genomes generated in the present study). Locally acquired cases recorded in October were circled by Cyan box.

The result is further supported by our phylogenetic analysis that the locally acquired cases in October are phylogenetically distant from the previous three waves. These new locally acquired cases are more closely related to the imported cases from India and Nepal (Figure 1). Our results suggest that the new spike in local infections may be triggered by a sudden surge of imported infections in late September, instead of hidden cases in the community leftover from the previous wave. More disturbingly, some locally acquired cases with the new viral genome did not have apparent epidemiological linkage and were geographically distant from each other (Supplementary Figure 3), indicating that the newly imported GH clade SARS-CoV-2 is currently disseminating in our community. This might be a warning sign that a fourth wave of COVID-19 infections is looming in Hong Kong soon.

Like the first three waves of infections, imported cases played a key role in spreading the virus. Currently, passengers from high-risk places (e.g. India and Nepal) must be tested negative for a SARS-CoV-2 nucleic acid test with specimens collected within 72 h before departure to Hong Kong, and are mandated to submit their deep-throat saliva (DTS) samples for another SARS-CoV-2 nucleic acid test upon arrival at the restricted area in the Hong Kong International Airport. They are required to wait for their test results at the holding centre before they can proceed with immigration procedures and subsequent compulsory quarantine for 14 days at their designated places in Hong Kong if tested negative [8]. Passengers arriving from areas with lower risk can proceed to their designated place for the 14-day compulsory quarantine after collecting their DTS samples at the airport [9]. Before the completion of the 14-day compulsory quarantine, they have to collect their DTS samples again and hand in their specimens either through their family members, friends or a door-to-door specimen collection service [10].

We suspect that the recent episode of local infections is attributed to undetected imported infections at the border. Although electronic wristbands are arranged to monitor travellers’ location during the 14-day compulsory quarantine, they can still be visited by their families or friends at their homes or hotel rooms. This could be a possible transmission route for the imported infections to the community.

As many parts in the world are experiencing a new wave of infections with exponentially increased confirmed cases, the city’s border control measures are needed to be strengthened to prevent imported infections. It would seem advisable to elevate the testing and quarantine arrangement for passengers from low-risk places to the same level as for those from high-risk places. During the 14-day compulsory quarantine, travellers would be better to stay in the quarantine centres or hotels designated by the Government, where visitors are not allowed.

Of particular concern is a recent much-touted plan to create “travel bubbles” because reopening the border would result in importing the virus once again. If there are more imported cases that slip through the net and the local transmission chains begin to coalesce, the scale of the outbreak can be catastrophic especially when they combine with untraceable cases that still circulating in the city.
